# The potential of programme theory in bridging the evidence into practice gap in implementation science: a worked example of integrating palliative care into heart failure management

**DOI:** 10.1186/s12913-025-12779-6

**Published:** 2025-04-30

**Authors:** Tracey McConnell, Carolyn Blair, Joanne Reid, Geoff Wong

**Affiliations:** 1 School of Nursing and Midwifery, Queen’s University Belfast, Belfast, UK; 2 Nuffield Department of Primary Care Health Sciences, University of Oxford, Oxford, UK

**Keywords:** Realist approach, Programme theory, COM-B, Behaviour change, Implementation science, Palliative care, Heart failure

## Abstract

Implementation science has been defined as the scientific study of methods focused on promoting the systematic uptake of research findings into routine practice in order to improve the quality and effectiveness of healthcare services. However, a recent critique of the science has highlighted a research to practice gap paradox suggesting that rather than closing the gap, implementation science may be reinventing it. Others have more recently provided a further critique focusing on the promises and pitfalls of implementation science, arguing that the field needs to further develop and grow. Our paper aims to contribute to this call by suggesting one possible way forward. To do this we first explore the ideas and assumptions underpinning implementation science, before we introduce the benefits of realist approaches - which are increasingly recognised as crucial for addressing the research to practice gap paradox as they deal with real world complexity. Whilst realist approaches may help move the field of implementation science forward, we also point out that the theories within this field can also help to improve understanding in realist research. In summary, our paper challenges the growing reliance on implementation theories, models and frameworks as the only starting point for research in this field, and we argue that realist programme theory may be equally useful.

## Contributions to the literature

- Currently a plethora of implementation theories models and frameworks exist, many of which list factors that influence implementation (theory of action), without explaining ‘how’ they influence implementation outcomes (theory of change).
- Realist approaches, by developing programme theory, can help overcome this limitation as they tell us what may influence implementation outcomes (theory of action) and how (theory of change).
- Implementation theories, models and frameworks may also constructively contribute to realist approaches, for example by providing a framework for organising findings or contributing explanatory concepts.
- Combining existing implementation theories, models and frameworks with programme theory can strengthen the explanatory power of realist approaches and provide implementation findings that are more useful to knowledge users.

## Background

### Ideas and assumptions underpinning implementation science

Historically, getting research into practice was viewed as a linear process, of developing an intervention, testing that intervention via a randomised controlled trial, and then implementing the intervention into a health service that embraced evidence-based practice [1]. This linear way of thinking about how the world works has been governed by the randomised controlled trial (if we prove it works, they will use it), without consideration of the complexity of the world and healthcare systems we operate in, where systems are embedded within systems, within systems [2]. It is unsurprising then that the research-practice gap has been likened to a ‘chasm’ [3].

Within the last few decades, the limitations of this linear view of implementation have been recognised, with the emergence of systems thinking, which is increasingly applied to healthcare [4]. Systems thinking views systems as outwardly distinct but codependent components (equipment, people, technologies), that have ever shifting and recurring behaviour patterns [5]. Complexity science also challenges the linear approach to implementation, that traditionally focused on only studying parts of a complex system (the people involved, the intervention of interest, and the anticipated outcomes) [6]. This narrowed and linear approach aimed to control for, or remove confounding variables that may disrupt cause and effect [7].

Healthcare systems are an example of a complex adaptive system (CAS) which (amongst other things) is made up of individuals with free agency, and are therefore unpredictable. The people within that system are interconnected, and therefore their actions change the context for other people in the system [8]. Although implementation science has emerged more recently in an effort to close the evidence to practice gap prevalent in healthcare, it has been criticised as being ‘antithetical’ (p.6) to complexity science, as it is underpinned by a reductionist way of thinking [1]. However, Braithwaite et al. [1] have also argued that, despite their different theoretical underpinnings, they can still be beneficial when used together to improve healthcare systems and practice. Nilsen [9] outlines the different theoretical approaches to implementation science such as process models (focus on guiding implementation process); determinant frameworks, implementation and classic theories (explaining what influences outcomes); and evaluation frameworks (for evaluating implementation efforts). Given the prolific growth in implementation Theories, Models and Frameworks (TMFs), Wang et al. [10] recently conducted a systematic review to appraise their usability, applicability, and testability. Wang [10] identified 143 TMFs which were judged to be limited across these outcomes. Frequent limitations included: the: lack of clarity around causal relationships among components of the TMFs; lack of practical guidance on how to apply TMFs; and lack of explicit hypotheses during the development stage of the TMFs.

Nilsen and others [9, 11] also highlighted the limitations of these implementation TMFs, as they do not provide *all* the key ingredients needed to translate evidence into practice, such as the understanding as to ‘how’ and ‘why’ an evidence-based intervention is likely to be implementable in different circumstances to achieve desired outcomes. Sarkies et al. [11] argues that implementation science is still guilty of studying parts of the implementation problem, such as context and mechanisms of change, as distinct variables which can provide the required steps for successful implementation. This checklist approach [11] suggests that varying contexts need to be controlled for, rather than seeing them as a normal part of a complex adaptive system [1]. A recent critique of the science has highlighted a research to practice gap paradox suggesting that rather than closing the gap, implementation science may be reinventing it [12]. Beidas et al. [13] have more recently provided a further critique focusing on the promises and pitfalls of implementation science, arguing that the field needs to further develop and grow.

In the meantime, other researchers have been approaching the implementation challenge in a different way. An approach that is being increasingly used to make sense of complex healthcare interventions are realist approaches – namely realist reviews or synthesis [14] (these terms are synonymous) and realist evaluations [15]. Sarkies et al. [11] provides a clear argument for, and illustration of the benefits of using a realist approach to overcome some challenges faced by implementation science in relation to dealing with complexity, by identifying the interrelationships between causal mechanisms, the outcomes they produce, and the contexts that will support change. Healthcare interventions are complex interventions introduced into complex systems and realist approaches have been developed to specifically try to deal with the complexity of the social context that interventions are implemented into [16]. Realist approaches are interested in understanding causes for changes and use the concept of mechanisms. Within implementation science, mechanisms of change are defined as core functions of a change process (what an intervention aims to do) [6]. In contrast, mechanisms in a realist approach, are conceptualised as being hidden, context-sensitive causal forces. In other words, hidden mechanisms cause outcomes but will only be triggered in the right context [17]. This model of causation for outcomes of interest is captured in the heuristic: context (C) + mechanism (M) = outcome (O) [17, 18]. This is not an equation, but a reminder to realist researchers to think configurationally. In other words, to explain the cause for an outcome, it is important to consider what the causal process (mechanism) is and also when this mechanism is triggered (i.e. in which context). In health services research, these underlying mechanisms are most usefully conceptualised as the reasoning that goes on in people’s head (which we cannot see happening – hence are hidden) that motivates them to take action to *cause* a behavioural change (the observable outcome that we can see and measure), but which require the right context (e.g., do people have the capacity and/or opportunity to carry out the desired behaviour? ).

Building these explanatory CMO configurations uses a process known as retroduction [19]. This is a theory-based approach that focuses on identifying the underpinning causal mechanisms that explain an outcome, and the context conditions under which that mechanism is activated or suppressed [19]. As highlighted by Mukumbang and Wong [20], there are two key approaches to developing theory needed to explain implementation success or failure; (1) starting with a substantial theory to guide data collection and analysis, and (2) starting with the data collected in the intervention context in order to develop theories that help explain the how and why different intervention resources interact within the context to help explain the outcomes and strategies needed to improve implementation.

### Benefits of implementation TMFs for realist approaches

The first approach, moving from theory to data, is the most common approach in implementation science, making use of substantive and implementation theories TMFs [20]. Substantive theories run across different disciplines, but are specific to a particular phenomenon [15], such as theories of change from psychology [21, 22].These substantive theories can provide a wealth of research evidence on a particular topic, making them useful for different aspects of evaluation, such as guiding research questions [15], and showing how seemingly unrelated findings correspond to each other [23] to enhance the explanatory power of findings [24].

Some TMFs are also described as middle-range theories within the implementation science literature, such as The normalisation process theory (NPT), which focuses on implementation, and normalisation of a new complex intervention within healthcare [25]. Middle-range theories are expressed at a higher level of abstraction (more generalisable) but are close enough to the empirical data so as to permit empirical testing [15]. According to Pawson [16] middle-range theories do not aim to explain everything in one theory, but rather they aim to explain the many parts that make up the whole, and this may be why they provide an especially useful framework for explaining how many seemingly unrelated findings correspond to each other.

There are an increasing number of realist studies detailing the benefits of using Implementation Science TMFs within realist approaches [26–28]. Examples include Downey et al. [28] who used The Consolidated Framework for Implementation Research (CFIR) [29] as a coding manual for data extraction, and then combined the CFIR with The Capability, Opportunity, Motivation Behaviour model (COM-B), and closely related Theoretical Domains Framework [30] to theorise causal links at the individual behaviour and systems level within their health services realist evaluation.

Flynn et al. [26] used the National Health Sustainability Model (NHS SM) [31] and Normalization Process Theory (NPT) [32, 33] to aid their understanding of the contexts and mechanisms that influenced sustainability of a healthcare intervention within their realist evaluation. Dalkin et al. [24] conducted a systematic review of realist approaches combining NPT to explore how complementary the two approaches are, and the practicalities involved in combining them. They found eight published studies that demonstrated key benefits; guiding the development of CMOcs and increasing explanatory power of findings.

In our experience from a recent realist synthesis [23] we found the key benefit of using an implementation TMF was in providing an organising framework to show how seemingly disparate findings from our myriad of CMOcs corresponded to each other to help tease out the key implementation strategies. As a background, the issue our project was trying to address was that despite over two decades of research, evidence showing improved outcomes for people living with heart failure, their families and the wider healthcare system along with policy and guideline support, integration of palliative care into heart failure management remains difficult to achieve in practice [34]. To address this paradox, we conducted a realist synthesis of the literature aimed at identifying what works, for whom and in what circumstances when integrating palliative care (PC) into heart failure (HF) management [35] More information can be found in our published protocol [36], findings paper [23] and larger NIHR report [37].

Our realist synthesis produced 6 CMOcs and 30 sub CMOcs [23] grouped under 3 core clusters including: (1) Culture change; (2) Practice change and; (3) Organisational change. We sought to find a way to translate these complex findings into a more accessible format that was useful for informing policy and practice [23]. The COM-B Model of behaviour change [30] provided an understanding of what was needed to support the ‘behavioural target’ (desired outcome), and what ‘intervention functions’ (i.e. intervention strategies) were more likely to help bring about the required change. This is where the use of substantive theory [17] (theories that function in different fields) which some implementation science TMF can be considered to be, are extremely valuable for helping to further interpret or frame findings from realist research [24].

**Fig. 1:**
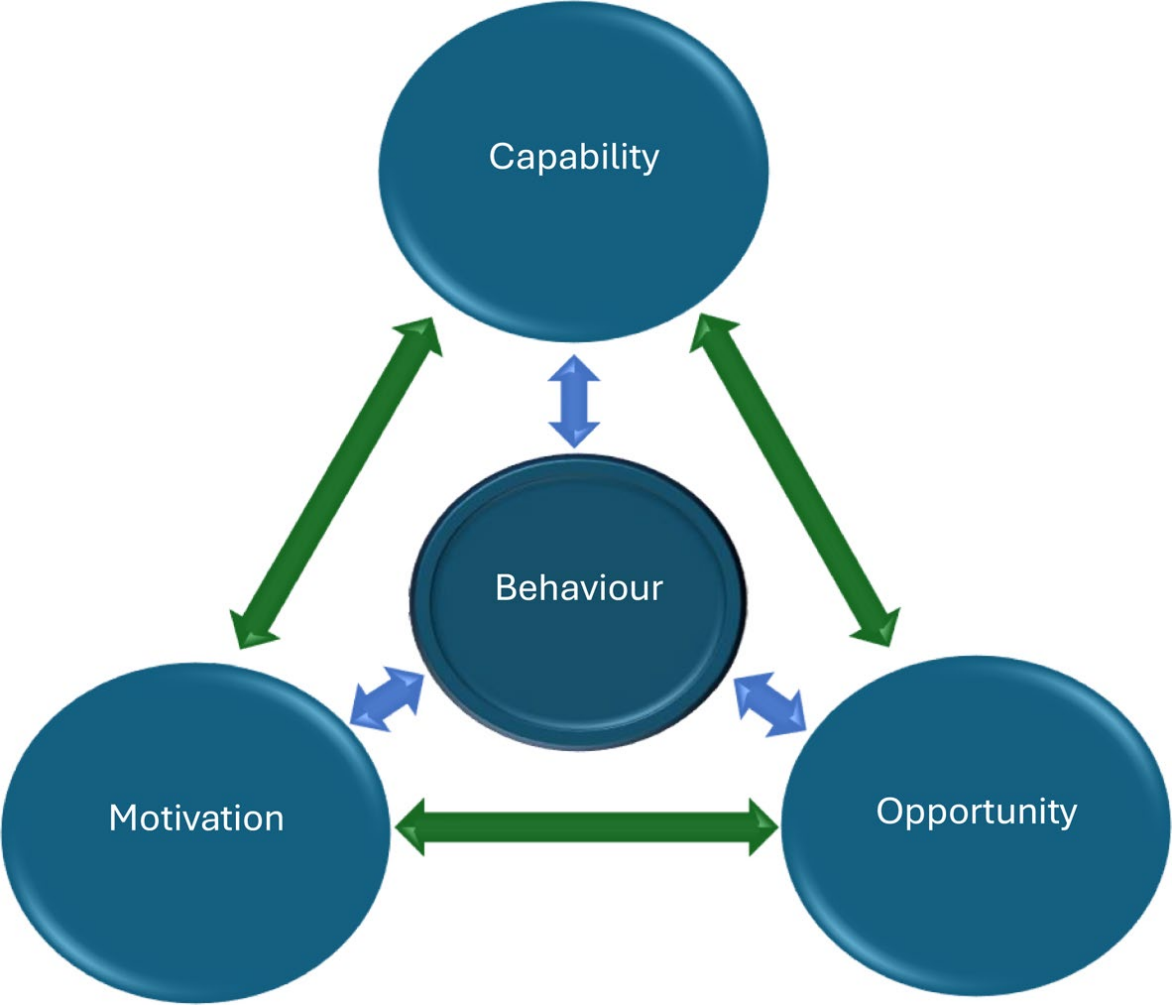
The core behavioural components of the COM-B model

The COM-B model classifies behaviour change into three main types: (1) Capability; (2) Opportunity and; (3) Motivation, visually detailed in Fig. 1. Capability refers to having the knowledge, skills and abilities to engage in a behaviour. Motivation refers to internal brain processes that influence behaviour. Opportunity refers to the external factors that enable or encourage a behaviour. The double-headed arrows in Fig. 1 emphasise the potential for each component in the system to influence another. Capability could influence motivation, and opportunity could influence capability. Furthermore, performing a behaviour could modify opportunity, capability and motivation.

**Fig. 2:**
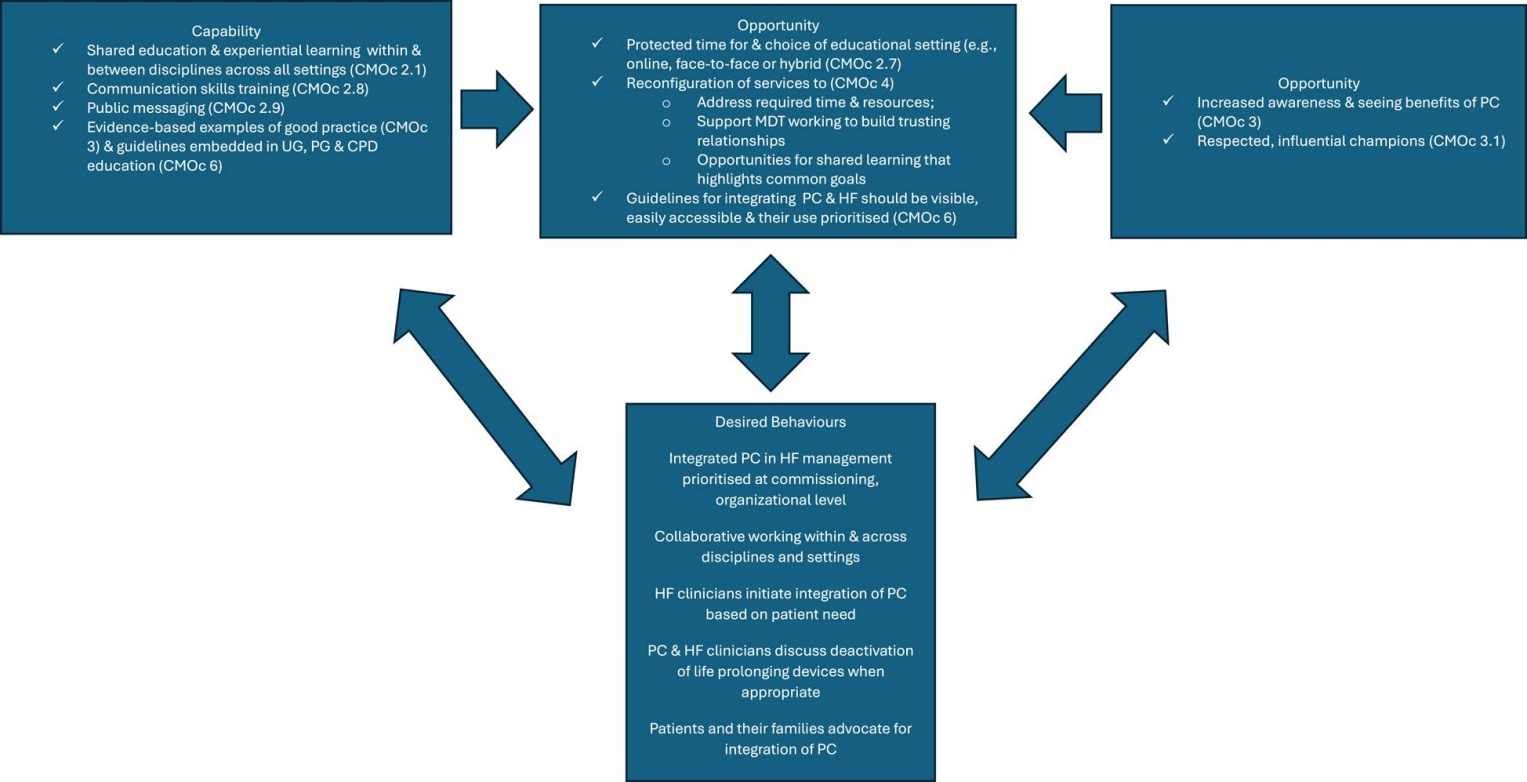
The COM-B model of behavior. Figure 2 reproduced from McConnell et al. [37]: An overview of intervention strategies likely to produce desired behaviours and avoid undesired behaviours to facilitate the integration of PC into HF management structured around the COM-B model. Abbreviations in Fig. 2: CMOc, context, mechanism and outcome configuration; COM-B, Capability, Opportunity, Motivation, Behaviour; CPD, continuing professional development; HF, heart failure; MDT, multidisciplinary team; PC, palliative care; PG, postgraduate; UG, undergraduate

Using these three core behaviour change requirements helped identify the intervention strategies that are likely to change the context to optimise implementation of integrated care (see Fig. 2). For example, the first cluster of CMOcs uncovered several ‘blockages’ and ‘flows’ that helped explain the difficulties of integrating palliative care and heart failure. In brief, there are subtle, but very powerful challenges imposed on integration of palliative care generated by the historical biomedical culture in which heart failure clinicians are trained and practice, which are reflected in an example CMOc below. The following sections adds to the discussion of previous work by McConnell et al. [23]

### Example 1

*When HF physicians’ and HF nurses’ training focuses predominantly on biomedical interventions to prolong life (Context)*,* they can be reluctant to consider PC (Outcome)*,* because they perceive they have failed in their care of the patient by doing so (Mechanism)*

This CMOc indicates a motivational issue, and as such we can draw on the COM-B model to help identify what strategies could help change this hindering biomedical culture that demotivates heart failure clinicians to integrate palliative care. For example, education that incorporates experiential learning with palliative care specialists could be used to help raise awareness and benefits of palliative care alongside heart failure treatment – thus increasing the chances of heart failure clinicians’ motivation to consider integrating palliative care.

### Example 2

The next illustrative CMOc is more about key stakeholder capability.

*When HF physicians and HF nurses have exposure to educational strategies that teach and prioritise PC (Context) they are more willing to provide generalist PC and know when to refer to or seek input from specialist PC (Outcome) because they have greater knowledge and confidence in their abilities to do so (Mechanism)*

For example, in terms of ‘capacity’, people need to believe they have the necessary knowledge and skills to carry out a required behaviour. This understanding of behaviour change helped identify education as a key intervention to address hindering contexts, such as a biomedical culture and misunderstandings that palliative care is synonymous with end-of-life care only. In turn, the realist synthesis of the literature helped uncover what ‘types’ of educational strategies are required to change the required behaviours, such as experiential learning; for whom change is required, e.g., those within palliative care and heart failure teams (capability), and how; seeing benefits of palliative care to increase motivation among heart failure clinicians to integrate palliative care into heart failure management.

### Example 3

However, according to the COM-B model, having capacity and motivation is not always enough to bring about the desired behaviour. The final key ingredient is having the necessary opportunity, that is the essential physical opportunities (such as time and resources) to support the desired behaviour change. Therefore, it is crucial to identify what intervention strategies are required to create the right opportunities, as reflected in the third example CMOc below.

*When physicians and nurses in HF and PC are given protected time and choice of educational settings (e.g.*,* online*,* face-to-face or hybrid) (C) they are more likely to attend (O) because they are empowered to do so (M)*

### Using realist programme theory to overcome the pitfall of having so many TMFs

Whilst the examples we have provided above illustrate the value of using implementation TMFs to better explain realist findings, the concept of programme theory from realist research can also be useful to overcome a pressing issue in implementation science. Namely, the challenge that a myriad of implementation TMFs have rendered the approach as complex as the process of implementation itself [38]. A scoping review by Strifler et al. [38] to identify implementation TMFs for evidence-based interventions to treat or manage chronic illness and cancer found 159 TMFs from 596 studies. Given that for a single topic area, researchers were using a large number of TMFs, it creates a challenge at the start of any research seeking to understand implementation – which TMF to use? This is where realist research may be able to provide some help by recommending that any research seeking to understand implementation might benefit from starting with programme theory. If we accept that a definition of programme theory is a ". description, in words or diagrams, of what is supposed to be done in a policy or programme (theory of action) and how and why that is expected to work (theory of change)” [15] then might not the theory of action (also sometimes called a theory of implementation) part of the programme theory suffice or be useful as a starting point to understand implementation?

Realist approaches are theory driven – because they set out to develop theory and use theory to make sense of data [39]. The theory they seek to develop is programme theory – which is a theory that explains how, why, in what contexts, for whom and to what extent interventions ‘work’. Programme theory is developed from primary data in a realist evaluation and from secondary data in a realist review [39]. For example, programme theory starts with the data collected in the intervention context in order to develop theories that help explain how and why different intervention resources interact within the context of the intervention to help explain the outcomes and strategies to improve implementation [20]. This is an example of the data to theory approach [20].

During a realist evaluation or review, programme theory is first developed and then iteratively tested (confirmed, refuted or refined) using data. Data are then analysed using a realist logic of analysis – i.e. using the heuristic C + M = O to develop context-mechanism-outcome-configurations (CMOcs). This realist understanding of causation is what makes realist evaluation and realist review unique. They move beyond using lists of factors that may support implementation, to include explanations of how and why outcomes are more likely to occur, in what contexts, and for which people. These explanations are encapsulated in a programme theory.

**Fig. 3:**
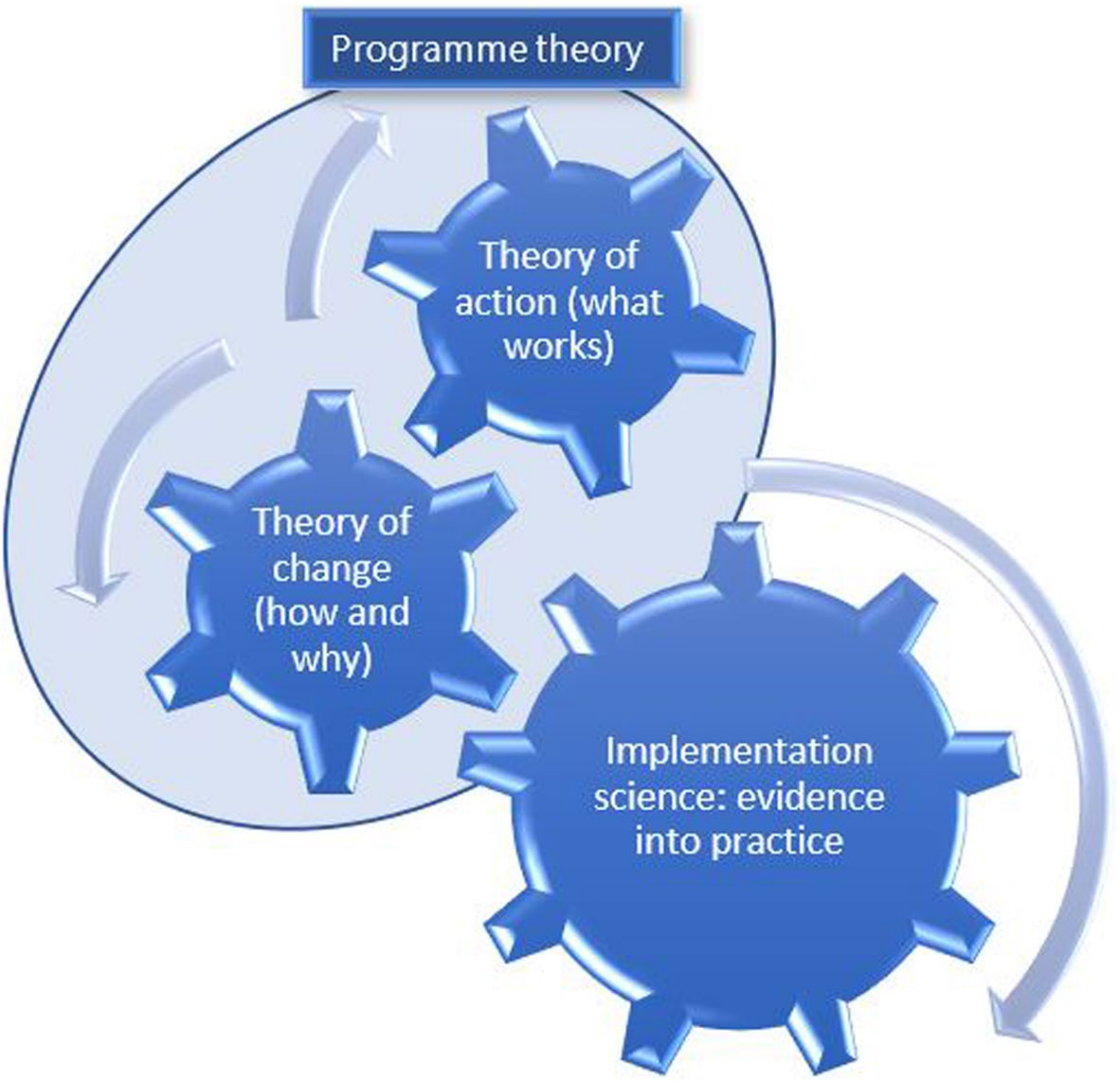
Realist programme theory: unique offering to implementation science for bridging the evidence into practice gap

The CMOcs provide both a theory of change and a theory of action. The theory of change is provided by the CMOc itself – as these explain when (context) and why (mechanism) an outcome occurs. By identifying the mechanism, CMOcs also surface for whom such an outcome would occur – this is because, as mentioned above, mechanisms are commonly responses within individuals in health services research and the ‘owner’ of any mechanism is also clarified. Understanding of the actions needed to get a desired outcome is clarified through the identification of the context that needs to be present for the outcome to occur. In realist reviews and evaluations, intervention strategies are used to manipulate context, so that the necessary context is present for a desired outcome to occur. Thus, CMOcs also provide an indication of how a desired outcome can be achieved – by elucidating what actions (i.e. intervention strategies) are needed. This clarification of which intervention strategies may be used to change context provides the theory of action. A realist programme theory brings together and organises CMOcs into a coherent explanation and thus provides both a theory of change and a theory of action in one theory (see Fig. 3).

Drawing again on our realist review on integrating palliative care (PC) into heart failure (HF) management [14], we used a data to theory approach to develop our initial programme theories, which then guided our eligibility criteria for included articles, data extraction and analysis.

## Discussion

Realist approaches have much to offer implementation science in terms of helping to close the evidence to practice gap which is so prevalent in healthcare. The realist understanding of causation, when captured within a programme theory, makes realist approaches unique as it enables explanations of how and why outcomes are more likely to occur, in what contexts, and for which people, rather than simply providing lists of factors that may support implementation, - thus addressing what has been highlighted as a major weakness attributed to implementation TMFs [10].

However, as also demonstrated in these examples, existing implementation TMFs can also have a useful role to play in realist approaches, providing useful understanding to guide actions needed to change contexts in such a way that desired outcomes are more likely to occur. Both add value and their combination is something that we suggest should be used more often.

While detailing the method and rationale for using an implementation science framework in a realist study is not in itself unique Dalkin et al. [24] did note that most authors did not reflect on their integration of the two approaches. This led them to stress the importance of appraising the theoretical and practical advantages of combining these approaches to advance knowledge in the field of implementation science. This is something we have done in this paper. In highlighting the value of starting with programme theory (data to generation of theory) and then combining an implementation TMF to provide greater explanatory power, we have attempted to add our appraisal of the theoretical and practical advantages of combining a realist approach with an implementation science model.

Although there are many benefits of using programme theory to advance implementation science by theorising the ‘how/why’ of implementation strategies, there are also a number of important considerations. The knowledge generated from the development of programme theory is fallible, depending on the interpretation of the research team as to what constitutes the causal mechanism of change, and in turn what constitutes the context responsible for activating that particular mechanism to produce a specific outcome. Furthermore, the programme theory, by trying to make sense of interventions used within a CAS, will at best only provide a partial ‘snapshot’ explanation in time – as CASs are in a constant state of flux [8, 40].

Therefore, as Pawson and Tilley [39] have stressed, we can only ever approximate the ‘truth’, about what will bring about a desired change, but we can get closer to a better understanding of how to optimise successful implementation by accumulation of our understanding of what works, for whom and in what circumstances over time. Therefore, the development of programme theory and implementation theories will always require further empirical testing [39].

## Conclusion

Implementation science theories, models and frameworks TMFs have helped advance the field. But there has been a proliferation of these TMFs, leading to researchers highlighting that there are now too many of them, with many having limited usability, applicability, and testability. In addition, many focus mainly on explaining what to do but much less so on how, why, for whom and in what contexts change happens [2–4].

A potential way forward to provide a simpler way to generate implementation findings that are more useful to knowledge users is to use realist approaches [5]. These develop realist programme theory - which encompasses both the theory of action and theory of change. Existing implementation science TMFs can still be incorporated into realist programme theory as they can help to deepen understanding or provide a useful framework. In this article we have provided a worked example of a realist programme theory that has been enriched by the incorporation of an existing implementation science TMF. The deployment of programme theory/realism is growing, but still emerging, and although there is a clear case for it, limited worked examples exist. We believe that this approach to implementation science holds promise, but more examples are needed to better understand the pros, cons and limitations better.

## Data Availability

Data from the realist synthesis used within this manuscript for illustrative purposes can be found in the published review.

## Abbreviations

TMFs: Theories models and frameworks
CMOc: Context-mechanism-outcome configuration
